# Transient hypertriglyceridemia: a common finding during Epstein-Barr virus-induced infectious mononucleosis

**DOI:** 10.1186/s12944-021-01603-9

**Published:** 2021-12-12

**Authors:** Emilio-Manuel Páez-Guillán, Joaquín Campos-Franco, Rosario Alende, Yago Garitaonaindía, Arturo González-Quintela

**Affiliations:** Department of Internal Medicine, Complejo Hospitalario Universitario, University of Santiago de Compostela, Santiago de Compostela, Spain

**Keywords:** Epstein-Barr virus, Infectious mononucleosis, Dyslipidemia, Hypertriglyceridemia

## Abstract

**Background:**

Hypertriglyceridemia can occur in lymphoproliferative disorders. Infectious mononucleosis is a self-limiting, benign lymphoproliferative disorder. This study aimed to investigate the serum triglyceride concentrations and their change over time in patients with infectious mononucleosis.

**Methods:**

We evaluated an adult patient with severe hypertriglyceridemia (>1000 mg/dL) during infectious mononucleosis and reviewed the records of 360 patients admitted to our hospital because of infectious mononucleosis (median age, 19 years; range, 15-87 years; 51.4% male). We compared the serum triglyceride concentrations with those of a control sample from the general population (*n*=75). A second triglyceride measurement, obtained during convalescence (median of 30 days after the initial determination), was available for 160 patients.

**Results:**

The triglyceride concentrations in the acute phase (median: 156 mg/dL) were significantly higher than those of the controls (median, 76 mg/dL; *P*<0.001). A total of 194 (53.9%) patients presented with hypertriglyceridemia (>150 mg/dL), which was more common in the patients older than 30 years than in the younger patients (78.6% vs. 50.6%; *P*<0.001). A significant correlation (*P*<0.005) was observed between the triglyceride levels and white blood cell counts, total cholesterol levels, and liver damage markers. The triglyceride concentrations decreased during convalescence (*P*<0.001) and were lower than the initial measurement in 83.7% of the cases. Conversely, the total cholesterol concentrations during the acute phase were lower than those of the controls and increased during convalescence (*P*<0.001).

**Conclusions:**

Patients with severe infectious mononucleosis frequently show mild, transient hypertriglyceridemia. Further studies are needed to elucidate the mechanisms underlying this finding.

## Introduction

Infectious mononucleosis produced by primary infection with the Epstein-Barr virus (EBV) is characterized by fever, pharyngotonsillitis, lymphadenopathy, blood lymphocytosis, and various potential systemic complications [1, 2]. EBV infects B cells, in which it induces polyclonal proliferation that is usually controlled by natural killer cells and the T cytotoxic response [3]. Infectious mononucleosis is an acute, self-limiting lymphoproliferative disorder [1]. Furthermore, EBV infection underlies certain malignant lymphoproliferative disorders, particularly in immunocompromised hosts [4, 5]. Few studies have investigated lipid disorders during infectious mononucleosis. Rivera et al. observed hypertriglyceridemia related to hepatitis in 6 cases of infectious mononucleosis [6]. More recently, Apostolou et al. observed atherogenic lipid changes in 29 patients with infectious mononucleosis, such as increased serum triglyceride (TG) concentrations [7]. Likewise, Sayyahfar et al. reported transient hypertriglyceridemia in 36 children with infectious mononucleosis [8].

Serum/plasma TGs are derived from 2 sources. Dietary lipids (exogenous TGs) are absorbed from the small intestine and enter the circulation stored in chylomicrons. Endogenous TGs are synthesized in the liver and released into the plasma in very low-density lipoproteins (VLDLs) [9]. The enzyme lipoprotein lipase (LPL) is the critical element that hydrolyzes TGs contained in chylomicrons and VLDLs, generating free fatty acids for peripheral tissues and regulating the plasma TG levels [10, 11]. Hypertriglyceridemia is a common lipid disorder in clinical practice, with an estimated prevalence of 10% in the adult population [9, 12]. An increase in the blood TG levels above 150 mg/dL is classified as moderate, whereas levels above 500 mg/dL are considered severe hypertriglyceridemia [13]. Hypertriglyceridemia can develop from a genetic disorder (monogenic or polygenic) or, more commonly, from secondary causes, such as diabetes mellitus (particularly type 2), hypothyroidism, renal disease, alcohol abuse, metabolic syndrome, obesity, excessive intake of long-chain TGs and free sugars, pregnancy, systemic lupus erythematosus, and certain drug therapies [9, 12, 14].

Herein, we report an adult patient with severe hypertriglyceridemia during infectious mononucleosis. We subsequently evaluated the serum TG concentrations, associated factors, and changes over time in a cohort of patients with severe infectious mononucleosis.

## Methods

### Design and setting

An index case with marked hypertriglyceridemia during infectious mononucleosis (see below) prompted the review of the clinical records of adult patients (15 years of age and older) with infectious mononucleosis who were admitted to the Department of Internal Medicine of the Santiago de Compostela University Hospital (Spain) between 1995 and 2018. The main reasons for hospital admission were severe signs of systemic inflammatory response, difficulty with oral intake, and the presence of complications. The hospital is a reference center for an area of approximately 400,000 inhabitants.

### Ethical approval

The study was reviewed and approved by the Galician Ethics Committee (code 2017/578) who waived the requirement for informed consent from the study participants, in agreement with Spanish regulations for retrospective studies of clinical records.

### Diagnostic criteria *(infectious mononucleosis)*

A definitive diagnosis of infectious mononucleosis was considered when the usual clinical syndrome was accompanied by positive immunoglobulin M (IgM) antibodies against the viral capsid antigen (VCA) of EBV and/or a positive heterophil antibody test [2].

### Study population

A total of 401 patients met the diagnostic criteria for infectious mononucleosis during the period. All the patients meeting the criteria with an available fasting TG measurement were included. The baseline (acute phase) serum TG determinations (measured the day after admission after 12 h of fasting) were not available for 41 cases. The index case reported in this study was not included in the analyses. Therefore, the study included 360 patients (51.4% male patients; median age, 19 years; interquartile range, 17-23 years; absolute range, 15-87 years). No patient was excluded for other reasons. Significant comorbidities were present in 25 (6.9%) patients and included bronchial asthma (4 cases), inflammatory bowel disease (3 cases), chronic seronegative arthritis (2 cases), type 1 diabetes mellitus (2 cases), cutaneous lupus (1 case), severe psoriasis (1 case), thalassemia minor (1 case), previous breast cancer (1 case), previous prostate cancer (1 case), previous Wilms tumor (1 case), epilepsy (3 cases), posttraumatic tetraparesis (2 cases), multiple sclerosis (1 case), congenital heart disease associated with Down syndrome (1 case), and ischemic heart disease (1 case). Four patients had a history of dyslipidemia, 3 of whom were undergoing atorvastatin therapy. The therapy for infectious mononucleosis before hospital admission and lipid determinations included antibiotics in 191 (53.1%) cases, nonsteroidal anti-inflammatory drugs (NSAIDs) in 135 (37.5%) cases, paracetamol (acetaminophen) in 131 (36.4%) cases, and corticosteroids in 25 cases (6.9%).

For 160 patients, a second TG measurement was available during the convalescence period (after a median of 30 days from the acute-phase determination; range, 14-177 days).

### Control population

A subsample of a study in the general population from the reference hospital’s health area [15] was used to compare the serum TG concentrations. The source study used an age-stratified random sample of the adult population from a single municipality belonging to the health area [15]. All the individuals aged 18-30 years from that study (*n*=75; 61.3% women; median age: 23 years) were included as a control population. None of the individuals were undergoing lipid-lowering therapy.

### Statistical analyses

The data were analyzed using IBM-SPSS Statistics for Windows software (24th version; IBM Corp., USA). Categorical variables were represented as absolute numbers and percentages. Given that numerical variables (age, hospital stay, serum TGs, TC, bilirubin, aspartate aminotransferase [AST], alanine aminotransferase [ALT], alkaline phosphatase, gamma-glutamyl transferase [GGT], and blood leukocytes [total count and differential lymphocyte count]) followed a nonnormal distribution (*P*<0.001 in the Kolmogorov-Smirnov test in each case), they were represented as medians and ranges, and nonparametric tests were used for the analyses. We employed the Mann-Whitney test to compare the numerical data between groups, and the Wilcoxon test to compare paired samples of numerical values. We used Spearman’s rank test to assess the correlations, and chi-squared test to compare the proportions. Two-tailed P values lower than 0.05 were considered statistically significant.

## Results

### Case report

A 60-year-old man was admitted to the hospital in 2003 because of fever (39 ºC) and jaundice. His symptoms had started 12 days earlier with general malaise, myalgia, and mild cough. His general practitioner had treated him with clarithromycin (500 mg bid) and paracetamol. The physical examination revealed only jaundice, the presence of some small cervical lymph nodes, and a palpable liver 2 cm below the costal margin. The main findings from the complementary examinations (Table 1) were the presence of blood lymphocytosis with abundant (>10%) atypical forms (activated lymphocytes), hyperbilirubinemia, and elevated serum transaminase. The patient also presented marked dyslipidemia, with a serum TG concentration >1000 mg/dL and total cholesterol (TC) >600 mg/dL, with increased low-density lipoprotein cholesterol (LDL-C), increased very low-density lipoprotein cholesterol (VLDL-C), low high-density lipoprotein cholesterol (HDL-C), and a low lipoprotein(a) [Lp(a)] cholesterol fraction, as measured by agarose gel electrophoresis (Table 1). The abdominal ultrasound highlighted only liver hyperechogenicity. Additional investigations revealed the following: weakly positive antinuclear antibodies in serum (1/80); IgG, 1580 mg/dL; IgA, 314 mg/dL; and IgM, 411 mg/dL (reference normal values: IgG 700-1600 mg/dL, IgA 70-400 mg/dL, IgM 40-230 mg/dL). The serological tests for human immunodeficiency virus, hepatitis B, and hepatitis C were negative. The tests for hepatitis A virus, herpes simplex virus, varicella-zoster virus, and cytomegalovirus revealed only a past infection (i.e., presence of positive IgG with negative IgM). The serum heterophil antibody test was negative; however, the IgM against the VCA of EBV was positive. In the subsequent weeks, the patient successively developed IgG antibodies against VCA, followed by antibodies against EBV nuclear antigen, with a progressive disappearance of IgM against VCA, confirming the EBV primary infection. In parallel, lymphocytosis progressively disappeared, the bilirubin and transaminase levels were normalized, and the serum lipid concentrations decreased (Table 1). After 17 years, with no specific treatment, the serum TG levels remained between 110 and 190 mg/dL, and the TC level between 220 and 250 mg/dL.

**Table 1:** Time-course changes of biological data in the index case

Day/ month	WBC count (/mm^3^)	Lymphocyte count (%)	Bilirubin (mg/dL)	ALT (IU/L)	Triglycerides (mg/dL)	Total cholesterol (mg/dL)	HDL-C (mg/dL)	LDL-C (mg/dL)	VLDL-C (mg/dL)	Lp(a)-C (mg/dL)	IgM-VCA	IgG-VCA	Anti-EBNA
**23/1**	10,240	67	9.9	207	360	ND	ND	ND	ND	ND	(+)	(-)	(-)
**27/1**	6300	73	7.1	183	367	697	18	583	93	3	ND	ND	ND
**05/2**	7420	59	3.8	143	1492	610	31	277	299	0	ND	ND	ND
**11/2**	6940	47	2.9	155	302	455	26	328	96	0	ND	ND	ND
**24/2**	7790	35	1.5	65	175	227	ND	ND	ND	ND	ND	ND	ND
**27/3**	6410	36	0.5	30	114	205	ND	ND	ND	ND	ND	ND	ND
**16/4**	7520	37	0.5	62	193	216	39	134	43	2	(+)	(+)	(-)
**03/6**	6000	38	0.3	36	147	242	ND	ND	ND	ND	(+/-)	(+)	(-)
**04/8**	8020	37	0.5	25	178	211	34	133	44	2	(-)	(+)	(+)

Cells counts were performed in venous blood. Biochemical determinations and microbiological studies were performed in serum

WBC, white blood cell count. ALT, alanine aminotransferase. VCA, Epstein-Barr virus capsid antigen. EBNA, Epstein-Barr virus nuclear antigen. HDL-C, high-density lipoprotein cholesterol. LDL-C, low-density lipoprotein cholesterol. VLDL-C, very low-density lipoprotein cholesterol. Lp(a)-C, lipoprotein-A cholesterol. Cholesterol fractions [HDL-C, LDL-C, VLDL-C, and Lp(a)-C] were measured by agarose gel electrophoresis. ND, no data available

(+), positive. (-), negative. (+/-), indeterminate value

The patient was treated with fenofibrate (600 mg bid, po) from 05/2 to 27/3

### Case series

**Fig. 1:**
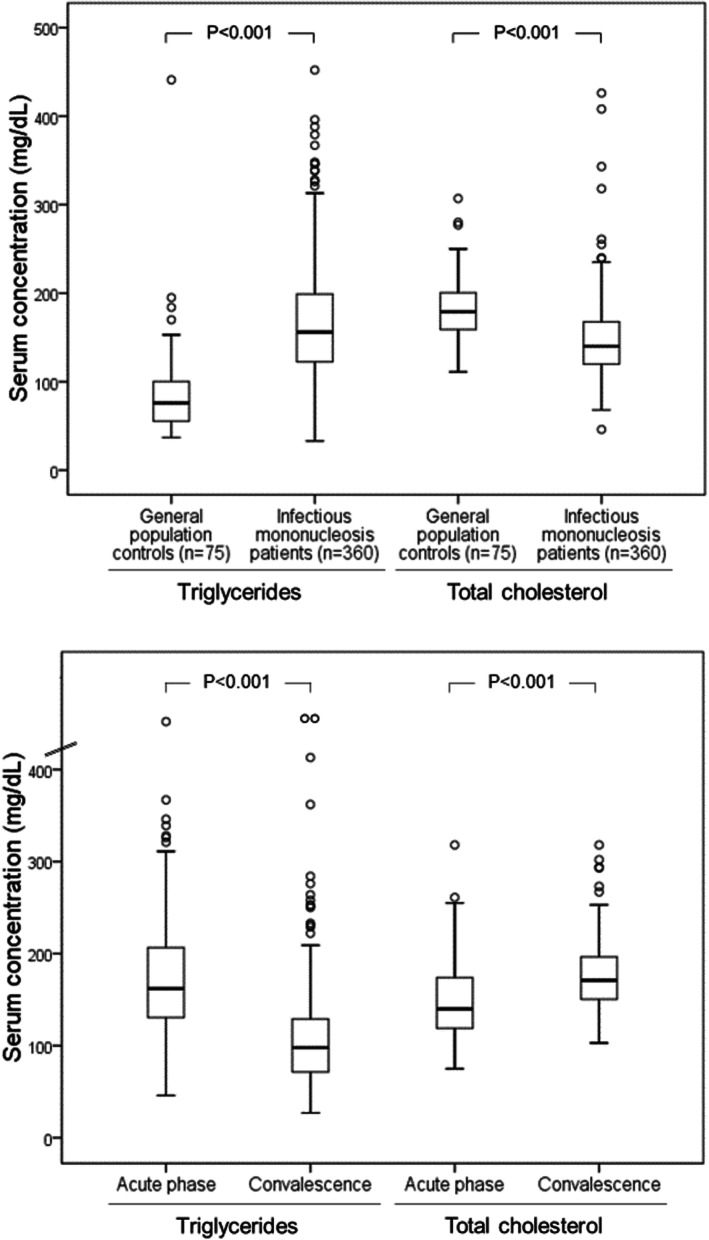
Upper panel: serum concentrations of triglycerides and total cholesterol in patients with acute infectious mononucleosis and general population controls. Lower panel: comparison of serum concentrations of triglycerides and total cholesterol in patients with infectious mononucleosis in the acute phase and the convalescence phase (*n*=160)

The median serum TG concentration during acute infectious mononucleosis was 156 mg/dL (range, 33-452 mg/dL). A total of 194 (53.9%) patients presented with hypertriglyceridemia (serum TG concentrations >150 mg/dL), and 18 (5.0%) patients presented TG concentrations >300 mg/dL. No patient presented complications that could be attributed to hypertriglyceridemia, such as hemolysis and pancreatitis. The serum TG concentrations in the patients with infectious mononucleosis were significantly higher than those in the healthy individuals from the general adult population (median, 76 mg/dL; range, 37-441 mg/dL; Fig. 1).

Table 2 shows the correlation between the serum TG concentrations and hematologic parameters, liver tests, and serum TC levels. A statistically significant positive correlation was observed between the TG concentrations and total leukocyte counts, lymphocyte counts, serum TC, bilirubin, AST, ALT, alkaline phosphatase and, particularly, the GGT levels (Table 2). A positive correlation between the serum TG concentrations and age was also observed (rho, 0.120; *P*=0.023). Hypertriglyceridemia (>150 mg/dL) was observed in 33 (78.6%) of 42 patients older than 30 years and in 162 (50.6%) of 318 patients aged 30 years or younger (*P*<0.001). No significant differences were found in the serum TG or TC concentrations among the patients taking or not taking antibiotics, NSAIDs, paracetamol (acetaminophen), and corticosteroids before admission (data not shown). A positive correlation was found between the serum TGs and hospital stay (rho, 0.141; *P*=0.008). The median hospital stays were 5 days (interquartile range, 3-7 days) and 6 days (interquartile range, 4-8 days) in patients with hypertriglyceridemia (TG >150 mg/dL) and patients without hypertriglyceridemia, respectively (*P*=0.003).

**Table 2:** Correlation between serum triglyceride concentration and hematologic parameters, total cholesterol and liver tests

	Blood lymphocytes (cells/mm^3^)	Blood leukocytes (cells/mm^3^)	Serum bilirubin (mg/dL)	Serum AST (IU/L)	Serum ALT (IU/L)	Serum GGT (IU/L)	Serum alkaline phosphatase (IU/L)	Total serum cholesterol (mg/dL)
**Coefficient**	0.121	0.164	0.296	0.206	0.166	0.387	0.303	0.354
**P-value**	0.022	0.002	<0.001	<0.001	0.002	<0.001	<0.001	<0.001

Correlation coefficients (rho) were calculated with the Spearman rank test (*n*=360)

AST, aspartate aminotransferase. ALT, alanine aminotransferase. GGT, gamma-glutamyl transferase

The serum TG concentrations significantly decreased during the convalescence phase (Fig. 1). Overall, the TG concentrations decreased in 134 (83.7%) of 160 patients. Serum TG concentrations were normalized in most of the patients in whom they were previously elevated (the second determination was normal [≤150 mg/dL] in 78.8% of 160 study patients).

The serum TC concentrations followed an inverse progression to that of TGs. In the acute phase of infectious mononucleosis, the TC levels were lower than those of the individuals in the general population. The serum TC concentrations increased significantly in the convalescent phase (Fig. 1).

## Discussion

This study showed that more than half of patients admitted to the hospital with infectious mononucleosis presented hypertriglyceridemia, which was more often observed in adults older than 30 years. Hypertriglyceridemia was mild in most cases, although 5% of the patients with acute infectious mononucleosis showed serum TG concentrations >300 mg/dL. Severe hypertriglyceridemia (TGs >1000 mg/dL) was observed only in the index case. The serum TG concentrations were correlated with leukocytosis, lymphocytosis, and liver injury markers. The hospital stay was longer in patients with hypertriglyceridemia (TGs >150 mg/dL) than in those without hypertriglyceridemia. Taken together, these results suggest that hypertriglyceridemia occurs in the most severe cases of infectious mononucleosis. The serum TG concentrations were significantly decreased in the convalescence phase. The serum TC levels followed an inverse course to that of the serum TGs, with low levels during acute illness that increased during convalescence from infectious mononucleosis.

Clinically, the results could have limited relevance, given that the TG elevation was mild (except in isolated cases) and generally transient. Furthermore, complications of hypertriglyceridemia such as pancreatitis and hemolytic anemia were not observed. Notably, however, acute conditions can affect certain laboratory determinations that, in turn, can have a relevant effect on patient management and prognosis, as has recently been observed in different settings [16]. In this regard, the possible long-term prognostic significance of hypertriglyceridemia during an episode of infectious mononucleosis is unknown. Normalization of the TG concentrations was not uniform, suggesting that infectious mononucleosis could aggravate preexisting dyslipidemia in certain cases.

Pathophysiologically, however, the results could be interesting because they can help explain certain lipid alterations in systemic disease. Infection and inflammation lead to multiple alterations in lipid and lipoprotein metabolism [17]. Sepsis can induce a decrease in the TC and HDL-C levels [18, 19]. Hypertriglyceridemia is usually less prominent in infectious and inflammatory diseases. The plasma TG levels can increase from increased VLDL secretion as a result of adipose tissue lipolysis, increased *de novo* hepatic fatty acid synthesis, and decreased LPL activity [17, 20]. In our study, lipid abnormalities in patients with infectious mononucleosis were similar to those observed in a detailed study of 29 patients of a similar age, including the high TG concentrations and low baseline concentrations of TC, HDL-C, and LDL-C [7]. Low concentrations of Lp(a) cholesterol were observed in the index case and are consistent with previous reports of low Lp(a) in mononucleosis patients [7] and in inflammatory states such as burns and sepsis [21]. Apostolou et al. also observed that increased TG levels at diagnosis were positively associated with the concentrations of inflammatory markers such as serum C-reactive protein and interleukin-1b. The authors also found a significant decrease in the baseline concentrations of apolipoprotein C (apoC)-III, a powerful inhibitor of LPL levels, compared with controls and a subsequent increase after 4 months of follow-up in the convalescence phase. No changes were observed in the apoC-II levels [7]. The activity of LPL is closely regulated, requiring different activators and inhibitors [11, 22, 23]. ApoC-II is necessary for LPL activation, acting as a cofactor [11], and severe hypertriglyceridemia has been reported in cases of apoC-II deficiency [11, 22, 23]. Conversely, apoC-III [23] and angiopoietin-like 3 [24] are inhibitors of LPL-mediated lipolysis. The increase in apoC-III inhibits LPL activity, increases the hepatic synthesis of TG-rich lipoproteins, and reduces their clearance [25]. Notably, the lipid pattern observed in infectious mononucleosis (a self-limiting, benign lymphoproliferative disorder) is similar to that observed in malignant lymphoproliferative disorders such as lymphoid leukemia (both acute and chronic) and non-Hodgkin’s lymphoma, in which the characteristic pattern includes hypertriglyceridemia, elevated VLDL, and low levels of HDL [26–31]. This pattern has been compared with that of LPL deficiency [30]. Furthermore, lipid abnormalities can precede the diagnosis of certain malignant lymphoproliferative disorders for which they could be markers of risk [32] and a marker for diagnosis and management [33]. Hypertriglyceridemia is also common in hemophagocytic syndrome [34], is a diagnostic criterion for the syndrome and is a marker for follow-up [35]. The so-called *lipid paradox*, which includes a contrast between a very low TC and/or HDL-C level and concomitant hypertriglyceridemia, has also been observed in certain forms of virus-induced hemophagocytic syndrome [36] and in additional immune-based disorders [37, 38].

## Study strengths and limitations

The limitations of the study are inherent to its observational and retrospective design. Retrospective studies have an inferior level of evidence compared with prospective studies because they are prone to confounding, selection and misclassification bias [39]. Stringent and validated selection criteria were used to minimize selection bias, but all the patients in this study were admitted to the hospital; therefore, the findings can only be applied to patients with infectious mononucleosis of similar severity. Given that charts were not originally designed to collect data for research, some information could have been missing. Additionally, a lack of homogeneity develops when different people are involved at different times in patient care, particularly concerning studies of cases that occurred over many years [39]. The availability of follow-up in most of the cases (retrospective cohort design) could be considered a strength of the study. However, the reasons for lost follow-ups often could not be ascertained and could potentially bias the results. The cholesterol fractions and apolipoprotein concentrations were not systematically measured and could not be analyzed in this series. Furthermore, the estimation of cholesterol fractions using classical formulas such as Friedewald’s has limitations in patients with hypertriglyceridemia [40]. Regarding the controls, a series of patients admitted with infectious processes other than infectious mononucleosis were not available for comparison purposes. Retrospective studies remain a useful tool to describe new manifestations and outcomes of disease and can form the basis on which prospective studies are planned [39]. However, retrospective studies generally only establish associations. Overgeneralization and claims of cause-effect relationships should be avoided in this type of study [39].

## Conclusions

In our study, patients with severe (in-hospital) infectious mononucleosis frequently showed mild, transient hypertriglyceridemia, which was more common in the patients older than 30 years than in younger patients. A significant correlation was observed between the TG levels and white blood cell counts, TC levels, and liver damage markers. The TG concentrations decreased during convalescence. Conversely, the TC concentrations during the acute phase were lower than those of the controls and increased during convalescence. Further studies are required to determine the mechanisms of dyslipidemia during infectious mononucleosis and its potential comparison with that occurring in lymphoproliferative disorders.

## Data Availability

The data are available from the corresponding author upon reasonable request and agreement with Spanish regulations for the use of clinical data.

## Abbreviations

AST: Aspartate aminotransferase
ALT: Alanine aminotransferase
EBV: Epstein-Barr virus
GGT: Gamma-glutamyl transferase
HDL: High-density lipoprotein
HDL-C: High-density lipoprotein-cholesterol
LDL: Low-density lipoprotein
LDL-C: Low-density lipoprotein-cholesterol
Lp(a): Lipoprotein(a)
LPL: Lipoprotein lipase
TC: Total cholesterol
TG: Triglyceride
VCA: Viral capsid antigen
VLDL: Very low-density lipoprotein
VLDL-C: Very low-density lipoprotein-cholesterol
